# Flexural Response of Degraded Polyurethane Foam Core Sandwich Beam with Initial Crack between Facesheet and Core

**DOI:** 10.3390/ma13235399

**Published:** 2020-11-27

**Authors:** Gurpinder Singh Dhaliwal, Golam M. Newaz

**Affiliations:** Department of Mechanical Engineering, Wayne State University, Detroit, MI 48201, USA; gnewaz@eng.wayne.edu

**Keywords:** core-facesheet debonding, CFRP/foam sandwich beam, bending behavior characterization, failure analysis

## Abstract

Structural systems developed from novel materials that are more durable and less prone to maintenance during the service lifetime are in great demand. Due to many advantages such as being lightweight as well as having high strength, corrosion resistance, and durability, the sandwich composites structures, in particular, have attracted attention as favorable materials for speedy and durable structural constructions. In the present research, an experimental investigation is carried out to investigate the flexural response of sandwich beams with a pre-cracked core-upper facesheet interface located at one end of the beam. During the development of the sandwich beams, an initial pre-cracked debond was created between the core and facesheet by placing a Teflon sheet at the interface. Both three-point and four-point flexural tests were conducted to characterize the flexural behavior of the sandwich beams. The effects of the loading rate, core thickness, and placement of the initial interfacial crack under a compressive or tensile stress state on the response and failure mechanism of Carbon Fiber-Reinforced Polymer (CFRP)/Polyurethane (PU) foam sandwich beams were investigated. It was found that the crack tip of the initial debonding between the upper facesheet and the core served as a damage initiation trigger followed by the fracture failure of the core due to the growth of the initial crack into the core in an out-of-plane mode. Finally, this leads to facesheet damage and rupture under flexural loadings. An increase in the core thickness resulted in a higher peak load, but the failure of the sandwich beam was observed to occur at significantly lower displacement values. It was found that the behavior of sandwich beams with higher core thickness was loading rate-sensitive, resulting in stiffer response as the loading rate was increased from 0.05 to 1.5 mm/s. This change in stiffness (10–15%) could be related to the squeezing of all pore space, resulting in the collapse of cell walls and thereby making the cell behave as a solid material. As a result, the occurrence of the densification phase in thick core beams occurs at a faster rate, which in turn makes the thick cored sandwich beams exhibit loading rate-sensitive behavior.

## 1. Introduction

Composite sandwich structures are developed by two thin but stiff, fiber-reinforced polymer laminated facesheets that are separated by relatively low-density material known as the core. Sandwich construction has attracted substantial attention in weight-critical applications, such as naval, aeronautical structures, high-speed marine craft, and racing cars due to its high stiffness-to-mass ratios, good flexural rigidity, manufacturing ease, excellent stability, and easily repairable [1,2,3]. Skins are adhesively bonded to the core in a sandwich composite construction. During the transfer of the load between the constituents, compressive loads are undertaken by one skin and the tensile loads are undertaken by the other, whereas shear loads are resisted by the core [4]. Due to this ability to withstand different loading conditions, the sandwich construction has favorable properties such as excellent stiffness, flexural rigidity, strength-to-mass ratio, and energy-absorbing capability [5]. It is at most important to have a strong bond between the core and the skins so that the structure can successfully withstand the shear and tensile stresses. The bond between the face sheets and core in sandwich structures may be undermined due to manufacturing defects and/or service loading, resulting in localized detachment between the face sheet and core (debonding) [6].

Localized debonding can also be a result of impact loadings, wave striking, and submarine explosions [6]. In the debonded sandwich structures, the tensile and shear loads are not efficiently transferred between the core and facesheet due to the presence of these defects; as a result, the load-carrying capacity of the structure is reduced. With the application of compression loading, the facesheet in the debonded structure may buckle over the locally separated area, resulting in the spreading of the separation defect and potential failure of the structure. Moreover, the debonding defects also significantly affect the fatigue lifetime and overall strength of sandwich structures subjected to static or cyclic loads [7,8]. Many researchers have extensively investigated the buckling behavior, failure mechanisms, and strength of such sandwich beams having facesheet/core debonding under compressive loads [9,10,11,12,13,14,15]. The manufacturing flaws and impact damage are represented by two-dimensional embedded debond geometries in sandwich structures. In the first category of research articles, debonding between the facesheet and core in sandwich columns is considered to be through-width debonding while studying the compressive behavior of such beams. For example, Avile et al. [9] developed an elastic foundation model for the analysis of the local buckling behavior of foam core sandwich columns containing a through-width face/core debond. They found that the Elastic Foundation Model (EFM) predicted the buckling strength of debonded sandwich columns with reasonable accuracy, although the predictions were unconservative. Vadakke et al. [10] conducted experimental research to study the in-plane compressive failure mechanism of sandwich specimens manufactured using glass/vinylester and carbon/epoxy facesheets over various Polyvinyl chloride (PVC) foam cores with an implanted through-width face/core debond. They reported that the buckling of the debonded facesheet followed by rapid debond growth toward the end of the specimen was the predominant failure mechanism in these specimens. Veedu and Carlsson [11] studied the buckling and collapse behavior of sandwich columns having a foam core and a through-width face/core debond using linear and nonlinear finite-element analyses. They reported that the buckling load is significantly affected by low foam densities and large debonds. In this study, higher buckling loads were provided by the linear analysis than the nonlinear analysis.

Sleight and Wang [12] investigated the behavior of the sandwich panel having two symmetrically located through-width debonds at the interface of the facesheet and core using finite-difference and finite-element analyses. In a realistic situation, debonding defects usually happen on one side of the sandwich construction due to production defects or impact loading. Thus, the consideration of symmetric defects is not an ideal representation of practical sandwich construction. Vadakke and Carlsson [13] carried out an experimental investigation to observe the importance of the faces and core in the failure patterns and maximum load withstanding capacity of the sandwich specimens composed of glass/vinylester face sheets and PVC foams subjected to compression loading. They observed that the compression failure of the facesheets was predominant for short specimens up to a gage length. Increasing the foam density increased this gage length, whereas decreasing the core thickness resulted in increased gage length. The anti-symmetric face wrinkling mode was dominant in the specimens having thick low-density core at intermediate gage lengths. The global buckling mode was the primary failure mode of long specimens. Moslemian et al. [16] examined the damage mechanisms of sandwich columns having an implanted through-width face/core debond subjected to compressive loads. In this research, the authors compared the fracture toughness data of specimens developed by conducting TSD (Tilted Sandwich Debond) tests to the energy release rate and mode mixity data. Sankar et al. [17] simulated the axial compression behavior of sandwich beams having a through-width debond between the facesheets and core using a nonlinear finite analysis technique. In the second category, the circular or square debond implanted in the middle of the sandwich specimen is considered.

Avilés and Carlsson [18] carried out research activity to study the localized buckling behavior of foam-cored composite sandwich panels with a face-core debond using three-dimensional finite element analysis. They also investigated the effect of core stiffness and debond size and shape. It was found that local buckling load is highly influenced by the core stiffness mainly for specimens having low modulus cores and smaller debonds. Sayyidmousavi et al. [19] studied the buckling behavior of laminated composite sandwich panels having face/core debond and transversely flexible core using the finite element method. They investigated the influence of geometric parameters such as the size, shape, aspect ratio of the debond, core stiffness, and fiber orientation of the face sheets on the buckling load of the panel. Different boundary conditions were enforced on the top and bottom face sheets of the panel in this study. The effect of circular-square interfacial debonding in the sandwich specimens developed by using Glass Fiber Reinforced Polymer (GFRP) as facesheets, PVC foams (H45, H100, and H200), and balsa wood as core subjected to in-plane compression by investigated by Aviles and Carlsson [6] by conducting an experimental study. They reported that the compressive strength of panels reduces with an increase in the debond size and decrease in core stiffness. The authors observed that panels with a circular debond that had a similar area to square ones failed at higher loads. Moslemian et al. [20] performed an experimental and numerical investigation to study the damage mechanisms of sandwich panels that had a predefined circular face/core debond and were subjected to compression loading. They reported that the energy release rate is hardly affected by the magnitude of the initial imperfection. At elevated loads, the effect becomes less dominant regarding the mode mixity. Thomson et al. [21] investigated the effect of interfacial crack and impact zone size on the shear characteristics and damage modes of sandwich composite beams developed from GFRP skins and a PVC foam core. They have reported that an abrupt decrease in the static shear strength of sandwich composite beams occurs when the interface crack size exceeds approximately 20–30 mm due to change in the damage mode from wrinkling of the facesheet to shear failure of core. The initiation of debond enhancement in foam-cored sandwich panels subjected to compression loading that had a circular face/core debond implanted at the middle of the panel was investigated by Aviles and Carlsson [22] through nonlinear finite element analysis. Chen and Bai [23] conducted finite element analysis to investigate the post-buckling response of the face/core debonded composite sandwich plate taking into account the matrix crack and contact effect. The authors stated that the matrix cracking failure and contact have a significant effect on the post-buckling behavior of the debonded composite sandwich plates. Avery and Sankar [24] studied the axial compressive response of debonded graphite/aramid honeycomb cored sandwich composites. Semi-empirical formulas for fracture toughness and maximum compressive load were developed from the test results. Some research investigations are also carried out on debonded sandwich structures to measure the accuracy of the developed analysis methods.

Berggreen et al. [25,26,27] studied the damage mechanisms of debonded sandwich panels subjected to non-uniform compressive and lateral pressure loading. They proposed a new numerical method for obtaining the mode-mixity at the crack tip. The mode mixity is very significant in the sandwich interface problems. Keeping this aspect in consideration, Berggreen [28] developed a 3D residual strength model that has a full account of mode mixity. Bull and Hallstrom [29] studied the behavior of curved sandwich beams that had a face/core debond subjected to a bending moment. A fundamental expression was developed by authors to have an easy assessment of an interface crack’s severity in a curved sandwich beam. The buckling response of a locally debonded facesheet layer from the core was investigated by Niu and Talreja [30] considering a Euler beam on the Winkler foundation with debonds subjected to in-plane compressive loading. They presented the analytical expressions of the buckling load and mode shape. The authors also reported the influence of length and situation of debonds on the ultimate load-carrying ability. Lindstrom et al. [31] carried out experimental research to observe the post damage behavior of sandwich panels that had symmetrically located edge debonds loaded with in-plane compression loads. Karaeva et al. [32] studied the interfacial shear strength (IFSS) of carbon fiber-reinforced polyurethane composites that had carbon nanotube grafting onto a surface of the fiber. They reported a 200% increase in the IFSS due to nanotube grafting. Shuai et al. [33] investigated the effect of surface modification of carbon fiber through silane molecules grafting on the CFRP/PU composites. They reported the improvement in tensile strength of CFRP/PU composites by 18.3%. Zhang [34] investigated the effect of 4,4′-diphenylmethane diisocyanate (MDI) molecules after electrochemical oxidation treatment on the tensile performance of thermoplastic polyurethane composites. Sanchez-Adsurar et al. [35] studied the influence of characteristics of carbon fiber on the performance of thermoplastic polyurethane-carbon fiber composites. Bin Hong and Xian Guijun [36] conducted a comparative analysis between a carbon fiber/polyurethane (CFRPU) and carbon fiber/epoxy pultruded plates for their resistance to sea water immersion. They reported that CFRPU plates performed superior to the epoxy and carbon fiber-reinforced plates. Sabina et al. [37] studied the hydrothermal aging behavior of a carbon fiber-reinforced laminate and its epoxy matrix in bulk conditions. Li Hao et al. [38] investigated the low velocity impact response of foam cored sandwich panels that had shape memory alloy hybrid facesheets. Liang et al. [39] conducted theoretical analysis on graded metal foam cored sandwich cylinders to investigate their performance under blast loading. Zhu et al. [40] studied the flexural characteristics of sandwich composite box beams subjected to three-point flexural loading. Hao Jingxin et al. [41] investigated the deformation pattern and damage characteristics of sandwich composite panels composed of wooden facesheets and Taiji honeycomb core subjected to three-point flexural loading. However, very limited articles are available in the literature characterizing the behavior of degraded/debonded sandwich beams that had facesheet/core debonding defects and studying damage propagation mechanisms in such composite sandwich structures under static three-point or four-point flexural loadings [42,43]. The high-order sandwich panel theory (HSAPT) model was used by Frostig and Thomsen [42] to investigate the nonlinear responses of debonded sandwich constructions loaded with three-point flexural loads that had a compressible core. They considered the delaminated zone free of shear stresses, which was situated at one of the face-core interfaces that had a through width crack. The partial contact and core’s transverse flexibility enabled the consideration of high-order effects in the model. Authors reported that delamination, which is of full contact type in nature, converts into a partial contact area along with the buckling of a compressed facesheet with the increase in the load. Mohsen et al. [43] performed experimental and numerical procedures to examine the influence of core-skin interfacial debonding on the response of sandwich beams under flexural loads. They reported that the location of embedded debonds on the tensile side had a negligible effect on the ultimate load-carrying ability of the sandwich beams, whereas the one on the compression side had significant effects.

**Figure 1:**
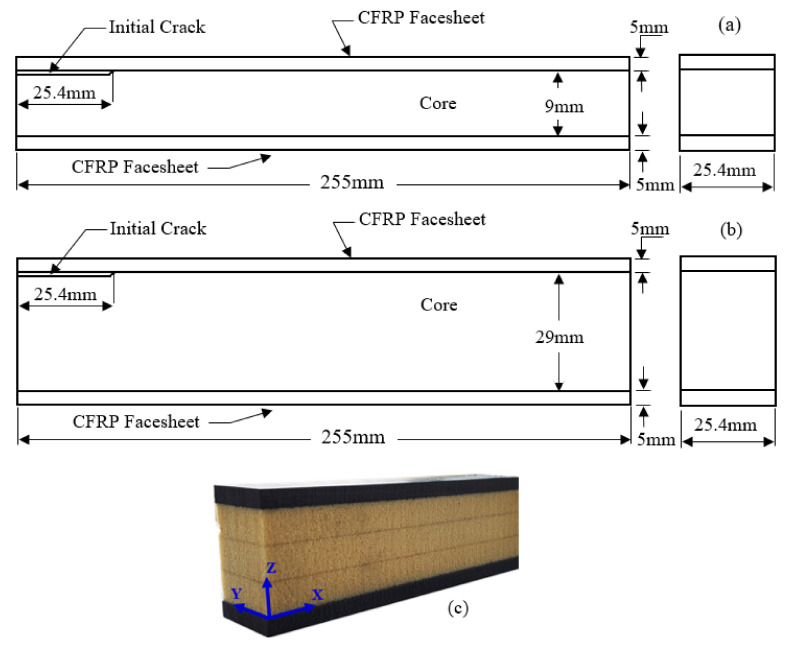
(**a**,**b**): Geometrical dimensions of different specimens studied; (**c**) Isotropic view of sandwich beam developed with thick Polyurethane (PU) foam core.

In the previous articles, the growth of an interfacial crack in the degraded sandwich beam is mainly studied under the compression and buckling loads, and these sandwich beam configurations with a face-core debond subjected to flexural loading have not been thorough fully observed and investigated. The study of degraded sandwich beams with interfacial defects is really important, as the bond is the only main source through which the acting loads is being transferred from a stiff facesheet to a core medium. Therefore, it is very crucial to investigate the debonding crack propagation in such degraded sandwich composites to have a clear understanding of their failure mechanisms for better maintenance under flexural loadings in general applications and primary load-carrying industrial structures. A comparative analysis of pre-cracked and normal beams is available in the literature; studying that will be just repetition of the subject with a different set of materials. So, this article is distinct, as it provides the comparative analysis for the behavior of such degraded sandwich beams under three-point and four-point flexural testing methods. It also studies the effect of different geometric parameters related to the sandwich beams on their response under these flexural tests. The exploratory test results that confirm the truth of modeling and give assistance to design methodologies are sparse in the publications. In the view of the foregoing investigations, the authors consider investigating the flexural response of CFRP/PU sandwich beams with a pre-cracked core-facesheet interface located at one end of beam (Figure 1). Such composite sandwiches mainly find their applications in the medical equipment such as the patient positioning table etc. due to their being lightweight, durable, transparent to X-ray radiation, having low attenuation, and having ease in machining to accommodate a specific insert for the attachment of medical instruments properties. Carbon fiber was selected in this composite because it reduces the intensity of X-ray beams rather than scattering the beams and risking unnecessary exposure to the patient. Polyurethane foam was selected due to its flame-retardant properties available in a range of densities. The usage of this composite in such a critical and precision oriented sector led to the motivation of studying the behavior of these degraded composites under flexural loadings. We are exploring how failure mechanisms related to interfacial debonding and related damage/degradation influence flexural behavior. Furthermore, the effect of loading rate, core thickness, and location of the initial debond on the response and failure mechanism of these sandwich beams were investigated. The novelty of this research lies in the fact that it shreds light on the flexural behavior of these beams under both three-point and four-point flexural testing methods that are vastly adopted by researchers.

## 2. Materials and Methods

The materials used in this research were woven carbon fiber/epoxy prepreg for the face sheet and polyurethane foam for the core, respectively. The polyurethane foam was supplied by MAG-ias Ohio, whereas the woven carbon fiber epoxy prepreg, known as VTM264/CF302, was manufactured by CYTEC (Kalamazoo, Michigan, USA). Each layer of the woven carbon fiber/epoxy prepreg had a 2 × 2 twill weave fabric style with 3 K FT300B40B fibers. Table 1 provides the in-plane mechanical properties of CFRP prepreg. Experimental tests such as tensile, compression, and shear tests were conducted at Wayne State University to determine these mechanical properties. Tensile, compression and shear tests on CFRP/epoxy laminate specimens were done as per ASTM D3039 [44], ASTM D3410 [45], and ASTM D5379 [46] test methods, respectively. In the tensile tests, the specimens were cut into 200 mm × 25 mm × 2mm dimensions with a 38 mm grip length on each side. Averaged stress strain data in both longitudinal and transverse directions were used to obtained moduli in both directions. For compression tests, thick CFRP/epoxy specimens of dimensions 150 mm × 25 mm × 4 mm were prepared with a 25 mm gauge length at the middle. End-tabs were employed in the gripping area of the samples. Global buckling of the specimens is reduced by using “Wyoming Compression Fixture”(Wyoming Test Fixtures Inc., Salt Lake City, UT, USA). Shear test fixture is employed on the MTS compression testing machine (Instron, Norwood, MA, USA) for the V-notched test. Thick test specimens were prepared as per the dimensions mentioned in the ASTM standard.

The polyurethane closed-cell foam used as the core in the sandwich construction has a material density of 248 Kg/m^3^. The mechanical properties of polyurethane listed out in Table 2 were also experimentally determined by conducting tension, compression, and shear tests. ASTM D1623 [47], ASTM D1621 [48], and ASTM C273 [49] were referred for conducting the tensile, compressive, and shear tests, respectively on polyurethane foam. In the tensile tests, Type A specimens were prepared as per the ASTM D1623 standard. Density values of the polyurethane foam were provided by the material supplier. The detailed geometric dimensions of specimens prepared to study the different aspects of these sandwich beams are illustrated in Figure 1. The sandwich panels were manufactured by stacking ten layers of woven carbon fiber epoxy prepregs on both the top and bottom sides of the polyurethane foam core to make the total thickness of each facesheet as 5 mm, as the thickness of each lamina was around 0.5 mm. An initial crack is implanted at the interface between the face sheet and core on one side of the sandwich panel by placing a 25.4 mm wide, 50.4 mm long, and 0.0762 mm thick Teflon sheet. During the manufacturing of sandwich beams, the interfacial voids are the main source of defects; thus, to represent those voids over a portion of the interfacial bond, such cracks are typically chosen. It is really hard to mimic the tiny voids with small interfacial implantation. Therefore, to make things easy, a simplification was adopted by incorporating the crack to a slightly greater area of interface bond. The location of the initial crack was chosen to be at the one end of the interfacial bond due to fact that under the flexural loading, the ends of the interfacial bond see higher shear stress, and a beam that has a majority of the its interfacial defects in that region will be most venerable in the performance. Thus, to study the worst case scenario, the implantation of the initial crack was selected to be at one end of the beam. The stacked sandwich panels were cured in the autoclave molding equipment (TMP) located in our laboratory that had the capacity to reach 350 °C temperature and deliver 350 KPa maximum pressure, respectively. During the curing process, the sandwich panels were treated under vacuum, and 344.7 KPa pressure was applied on the sandwich panel while maintaining them at 135 °C temperature for 20 min. Later, the sandwich constructions were cooled down by passing mist and water over the heated platen for 15 min each. The sandwich composite panels were post cured also in an oven by maintaining them at 80 °C for 5 h. The curing cycle parameters listed above are adopted based on the recommendations provided by the material suppliers i.e., CYCTEC and General Plastics. A resin diffusion was observed from the facesheet into the core for all composite panels during manufacturing. The thickness of diffusion from the actual facesheet and core interface was found to be approximately 0.5 mm with closer examination. The band saw with a special cutting blade designed for CFRP layers was used to cut the sandwich panels into specimens that had 255 mm length and 25.4 mm width for testing. The total thickness of the samples was 19 and 39 mm in different categories.

## 3. Experimental Setup

ASTM standards D790 and D6272 were referred to investigate the flexural response of sandwich specimens under both three and four-point flexural loadings. These are comparatively appropriate for this work, as these two closely depicts the short beam flexure configuration of (ASTM C393) [50], which is the Standard Test Method for Flexural Properties of Sandwich Constructions. Considering these similarities between both tests methods, the author believes both tests can be appropriately used in this study. Typically, flexural modulus, bending stress, and strain values are provided by the flexural tests. The four-point bending test is pretty much identical to the three-point flexural test. The crucial dissimilarity between these two flexural tests lies in the fact that the introduction of a fourth load-bearing point in a four-point test places a significantly higher portion of the beam under ultimate stress, contrary to bringing a very small region of the beam that is directly underneath the central load-bearing point. The shear and local deformation effects in the loading and the supporting portions of the beam are taken into account in the conventional four-point bending test method. The primary usage of this test method is to obtain the mechanical characteristics of materials primarily exposed to flexural forces. A major advantage of these flexural tests is that the specimen’s geometries are significantly easy to manufacture, and there are zero occurrences of gripping problems such as tensile tests in the current investigation; flexural tests were carried out using an MTS machine with a load capacity of 22 kip.

**Figure 2:**
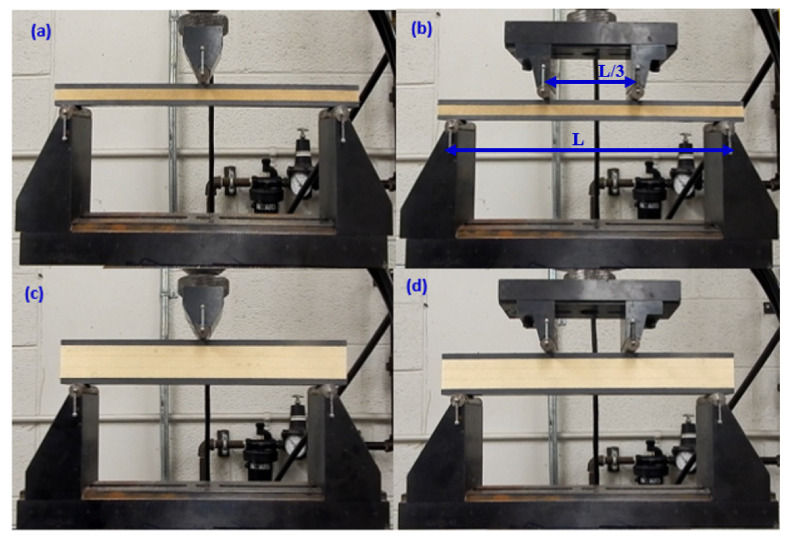
Three-point and four-point flexural test setup pertaining to specimens that had (**a**,**b**) a core thickness of 9 mm; (**c**,**d**) a core thickness of 29 mm.

Three and four-point bending fixtures developed by Wyoming test fixtures Inc. were utilized to perform these flexural experiments at room temperature. The specimen is loaded and supported with the cylindrical points that have a diameter of 12.7 mm in these fixtures. In the four-point tests, the compressive force is applied to the specimen at one-third point loading (L/3) to maintain the load-span as 76.2 mm and support-span as 228.6 mm. The support span length was varied in three-point bend tests to investigate its effect on the load-displacement and damage patterns. Figure 2a–d shows the experimental setup and different configurations of specimens in the course of the three and four-point flexural tests. The displacement control mode of the MTS equipment was chosen to quasi-statically conduct the flexural tests by moving the lower support point upward against the specimen. The flexural tests were performed at a rate of 3 mm/min. The acquisition system of the equipment was used to record the force, machine displacement, and time at intervals of 0.5 s.

In a particular category, five specimens were tested to obtain the scatter range and confidence bounds. Finally, the resultant test data were averaged and plotted into a single graph. We have also used such a program named “Lsprepost” to get the averaged curves from the all the tested specimens. To clarify again, we have obtained the averaged curves from the five curves of the tested specimens by finding out the average of every data point in those multiple curves at a particular time step as the time interval between successive steps, for the data acquisition was the same among them.

## 4. Results and Discussion

### 4.1. Effect of PU Foam Core Thickness

**Figure 3:**
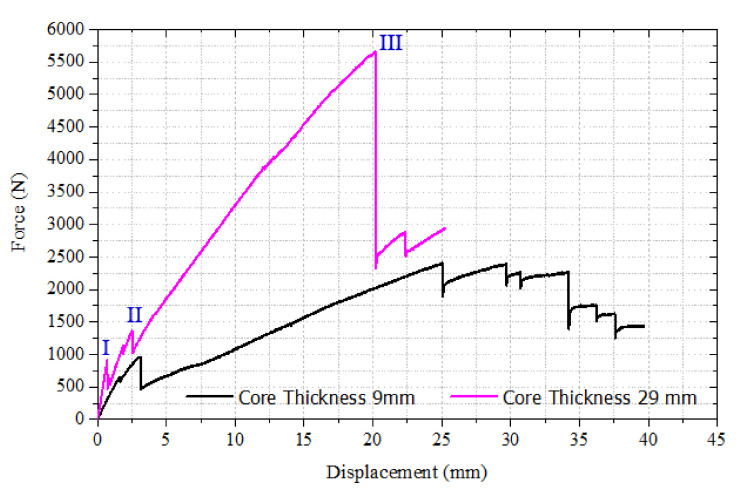
Effect of core thickness on Carbon Fiber-Reinforced Polymer (CFRP) facesheet/PU foam sandwich beam under four-point flexural loading at a displacement rate of 0.05 mm/s.

**Figure 4:**
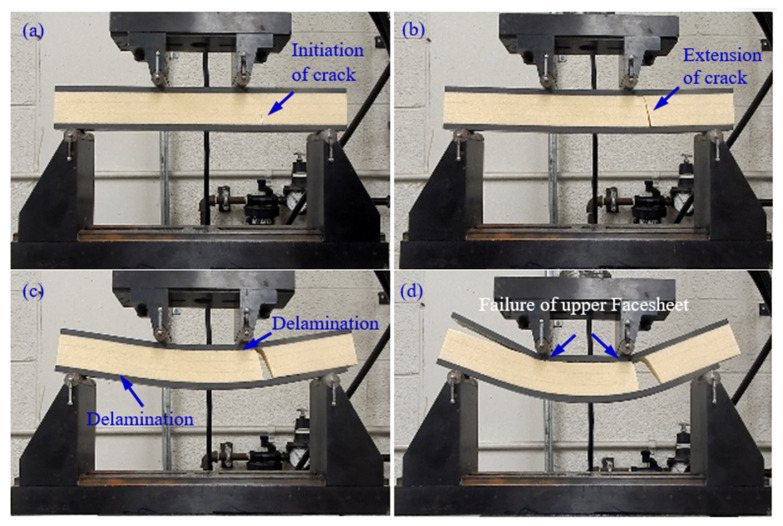
Damage morphologies of a CFRP/PU foam sandwich beam with a thick core under four-point flexural loading: (**a**) Initiation of crack; (**b**) Crack Extension; (**c**) Delamination; (**d**) Upper Facesheet Failure.

**Figure 5:**
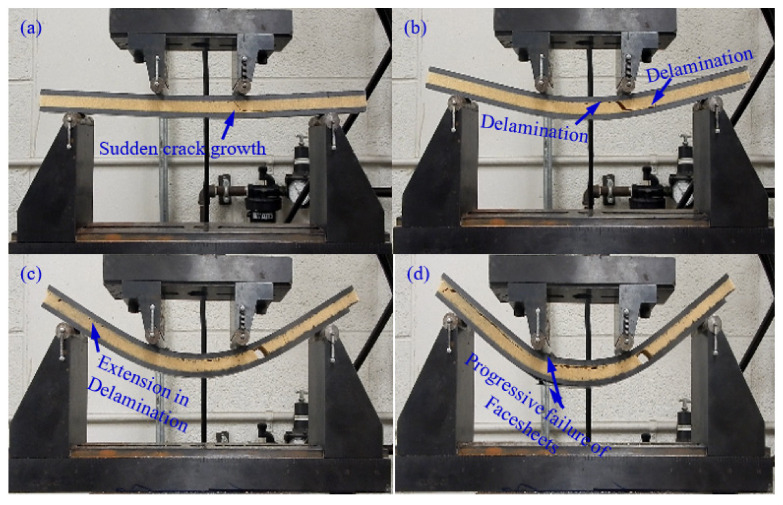
Damage morphologies of a CFRP/PU foam sandwich beam with a thin core under four-point flexural loading: (**a**) Crack occurrence; (**b**) Delamination; (**c**) Delamination Propagation; (**d**) Facesheets Progressive Failure.

The effect of polyurethane foam core thickness on the flexural behavior of a CFRP facesheet/PU foam sandwich beam under four-point flexural loading at a displacement rate of 0.05 mm/s, load span length of 76 mm, and support span length of 228 mm is illustrated in Figure 3. It was observed that the initial interfacial crack acts as the trigger for the initiation of the through-thickness crack in the PU core under the application of the flexural load, resulting in a small decrease in the stiffness of the beam as indicated by point I in Figure 3 and Figure 4a. With the increase in the applied load, the crack is extended further and reaches the upper facesheet, resulting in a further decrease in stiffness as shown in Figure 3 by point II and Figure 4b. With further advancement in the application of the flexural load, a delamination failure is observed between both the upper and lower facesheet and core, as illustrated in Figure 4c. Finally, the compressive rupture failure of the upper facesheet is observed, resulting in a significant decrease in the load levels as shown by point III in Figure 3 and Figure 4d. The peak force observed in the sandwich beam that had a thicker core was observed to be approximately around 5600 N, whereas the compressive rupture failure of the beam was observed at the displacement of 20 mm. The CFRP/PU foam sandwich beam that had a smaller core thickness (9 mm) also mainly shows the same flexural response as that of a sandwich beam that had a thicker core (29 mm) but results in a lesser peak load value (approximately 2500 N) and higher rupture failure displacements (35 mm) as compared to a sandwich beam that had a thicker core. Among the other distinctions in the behavior of both sandwich beams, it was observed the sandwich beam that had a less thick core shows the sudden and full crack growth through the core at the initiation of the crack only, as shown in Figure 5a. The sandwich beam with a smaller core thickness shows the progressive failure of the face sheets after the shear failure of the core illustrated by Figure 5d, whereas the thicker core sandwich beams show a compressive rupture failure of the face sheets after the shear failure of the core as illustrated by Figure 4d. In other words, the increase in the core thickness from 9 to 29 mm results in the momentous rupture of the upper facesheet as compared to progressive failure of the facesheet under four-point flexural loadings. The compressive strength of the CFRP/epoxy is smaller than the tensile strength due to which we see a compressive failure of upper facesheet which is under the compressive stresses rather the bottom facesheet, which is under the tensile stresses as shown in Figure 4d. The damage morphologies of the sandwich beam that had a smaller core thickness under four-point flexural loading are shown in Figure 5. Table 3 shows the peak force and maximum displacement of both thick and thin cored specimens under four-point flexural loadings. The minimal scatter in the data shows the repeatability of the data. It is hard to discuss the result with the literature, as only limited research is available on the topic that has some degree of similarity. The pattern of the results will be same even if we used a different set of materials for the facesheets such as glass fiber-reinforced epoxy laminates and PU foam core, but the results may change of the type of core is changed.

### 4.2. Effect Displacement Rate of Loading Pin

**Figure 6:**
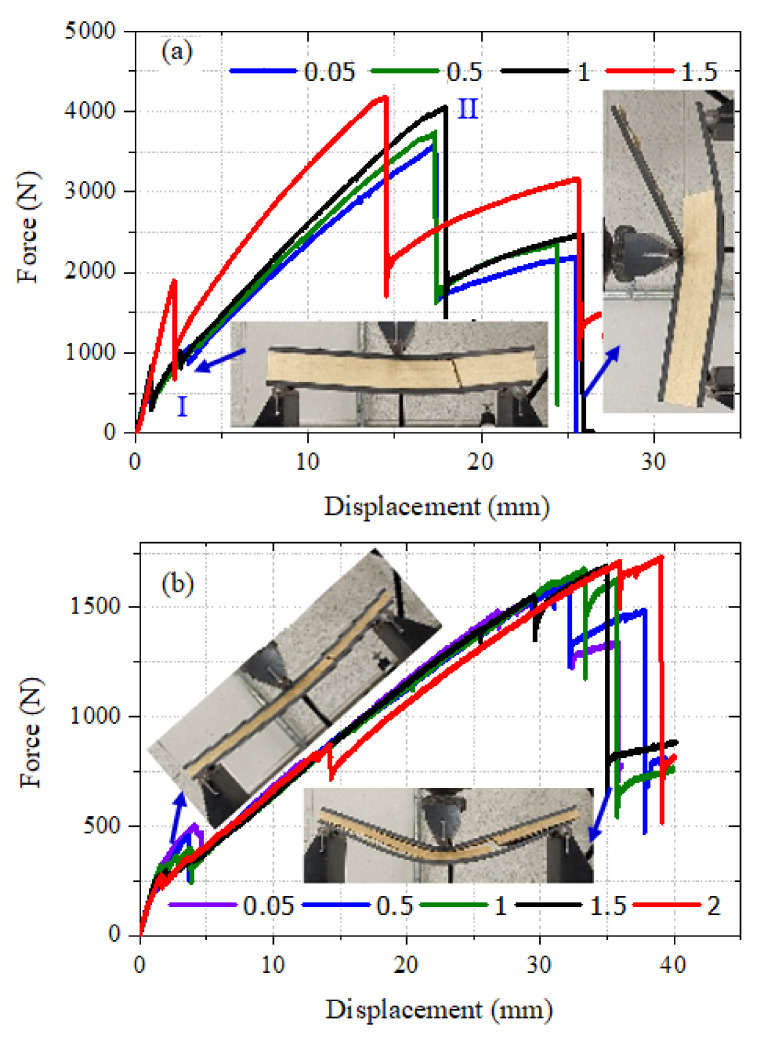
Effect of displacement rate on a CFRP facesheet/PU foam sandwich beam that had a core thickness under three-point loading at a span length of 9 inches; (**a**) 29 mm and (**b**) 9 mm.

The effect of the displacement rate of loading pin on the behavior of a CFRP facesheet/PU foam sandwich beam that had a core thickness of 29 mm and 9 mm under three-point loading at a span length of 9 inches was investigated. CFRP/PU foam sandwich beam specimens were tested at displacement rates of 0.05, 0.5, 1, and 1.5 mm/s. The force-displacement response of the sandwich beam that had a thick core tested at different loading rates is illustrated by Figure 6a; similarly, the behavior of the sandwich beam that had a thin core is presented in Figure 6b. It is viable from Figure 6a, that the sandwich beam that had a thick core showed a displacement rate-dependent behavior. This is important because CFRP/epoxy material is usually considered as non-strain-rate dependent material. Even the inclusion of a thinner core (9 mm) did not affect the overall nature of the sandwich composite under variable loading conditions; however, the inclusion of more polyurethane foam (29 mm) made the overall nature of the sandwich composite turn out to be strain rate-dependent because the volume fraction of the PU foam became increased as compared to the CFRP volume fraction in the sandwich composite. So, the above statement is made to mark out this difference in both types of sandwich composites. Higher peak load values were observed for this beam as the loading rate is increased. The beam tested at 0.05, 0.5, and 1 mm/s showed relatively similar behavior in terms of first core shear failure displacement and post-peak load failure displacement indicated by points I and II in Figure 6a. However, the sandwich beam tested at a 1.5 mm/s loading rate presented a comparatively different behavior.

**Figure 7:**
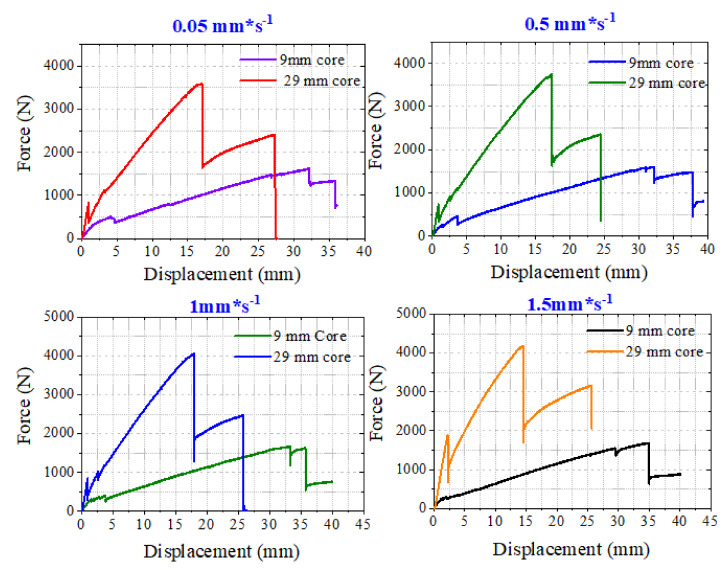
Comparative analysis of behavior of a CFRP facesheet/foam sandwich beam under three-point loading that had a span length of 9 inches at different displacement rates.

Relatively higher force and displacement values for the core shear failure instance were observed. In general, a stiffer response of the CFRP/PU sandwich beam was observed for a displacement loading rate of 1.5 mm/s. The displacement corresponding to the peak force and initiation of progressive damage phase was also on the smaller end as compared to beams tested at other loading rates. The CFRP/PU sandwich beam that had a thin core was not largely affected by the loading rates. The only minor effect was noticed in the force and displacement values corresponding to the initiation of the through-thickness crack in the core resulting in core shear failure at extended loads. It was noticed that the force and displacement level corresponding to the initiation of a shear crack in the core was reduced (force approximately 500 to 250 N and displacement approximately 5 to 2 mm) with the increase in the loading rate from 0.05 to 1.5 mm/s. The damage morphologies of both sandwich beams with thick and thin cores were not affected by the rate of displacement. The typical failure mode of the CFRP/PU sandwich beam includes through-thickness crack initiation in the core, resulting in shear failure of the core at extended load levels, delamination growth between the core and both upper and lower facesheets, and finally, the sandwich beam failing progressively due to the compressive failure of upper facesheet. The typical damage modes of the beams are shown in Figure 6a,b. A comparison between the behavior of thick and thin core CFRP/PU sandwich beams tested at different loading rates under three-point boundary conditions is carried out by plotting their response jointly in a single plot, as illustrated in Figure 7. At all the loading rates, the sandwich beam with a thick core shows higher peak force and lower failure displacement values than the sandwich beam with a thin core.

### 4.3. Effect of Placing the Initial Interfacial Crack under the Compressive and Tensile Stress Region.

**Figure 8:**
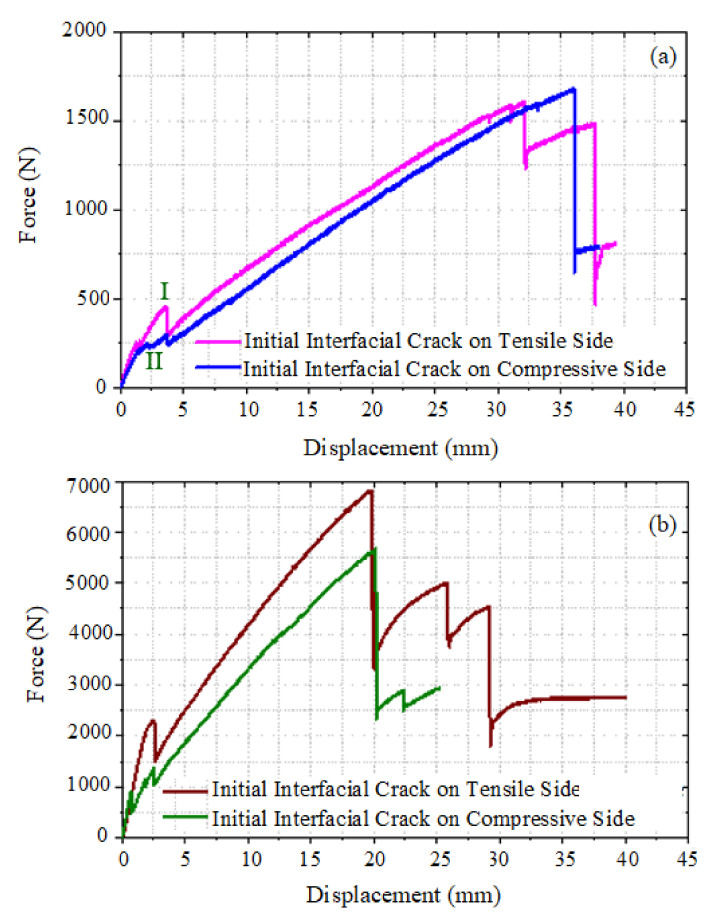
Effect of position of the initial interfacial crack, (**a**) CFRP/PU sandwich beam with a thin core tested under three-point loading, and (**b**) CFRP/PU sandwich beam with a thick core under four-point loading.

**Figure 9:**
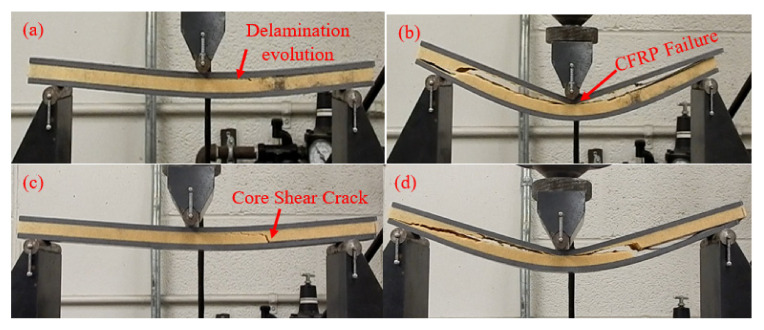
Damage morphologies of a CFRP/PU sandwich beam that had a thin core tested under three-point loading; (**a**,**b**) initial interfacial crack on the compression side; (**c**,**d**) initial interfacial crack on the tensile side.

**Figure 10:**
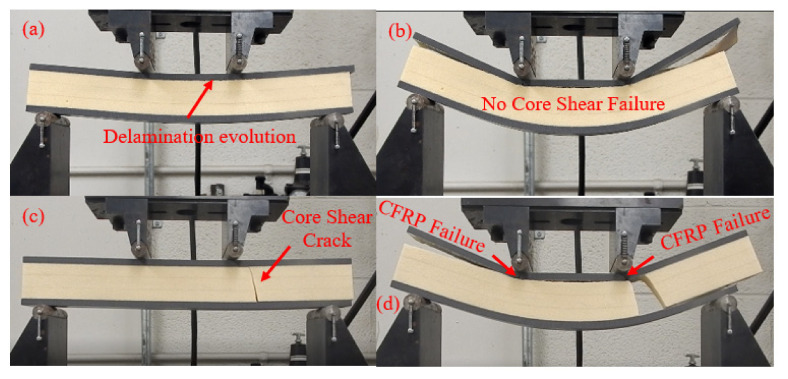
Damage morphologies of a CFRP/PU sandwich beam that had a thick core tested under four-point loading; (**a**,**b**) initial interfacial crack on the compression side; (**c**,**d**) initial interfacial crack on the tensile side.

The effect of placing the initial interfacial crack under compressive or tensile stress on the mechanical response of the CFRP/PU beam and damage morphologies was investigated. Sandwich beams with a thin and thick core, which are tested under three-point and four-point loading conditions, respectively, were used in this study. Figure 8a,b illustrate the effect of the location of the initial interfacial crack in CFRP/PU sandwich beams with a thin core tested under three-point loading conditions and a sandwich beam with a thick core tested under a four-point loading scenario, respectively. Sandwich beams that had an initial interfacial crack placed under the tensile stresses showed the stiffer response as compared to the beams that had an initial interfacial crack placed under the compressive stresses. The location of the initial interfacial crack also affected the damage morphologies of the beams. The presence of an initial interfacial crack on the compression side reduces the stiffness of the beam due to the evolution of delamination between the upper facesheet and core as shown in Figure 8a by the point I and Figure 9a, whereas positioning the initial interfacial crack on the tensile side triggers the core shear failure, resulting in stiffness reduction of the beam due to evolution of a through-thickness crack, as shown by point II in Figure 8a and Figure 9c. Compressive failure of the upper CFRP facesheet was observed as a damage mechanism at the final displacement loading increment in both types of beams that had a thin core. The comparative analysis of damage of the CFRP/PU sandwich beam that had a thick core tested under four-point loading by positioning the initial interfacial crack under compressive and tensile stress regime is illustrated in Figure 10. The damage mechanism of the thick core beam was almost identical to the thin core beam with the exception that it did not show the core shear failure when the initial interfacial crack was placed on the compressive side. Therefore, it can be concluded that additional core thickness does not allow the core shear failure to initiate and evolve; even the upper CFRP facesheet fails under compressive loading underneath the loading pins for specimens that had an initial interfacial crack on the compressive side.

**Figure 11:**
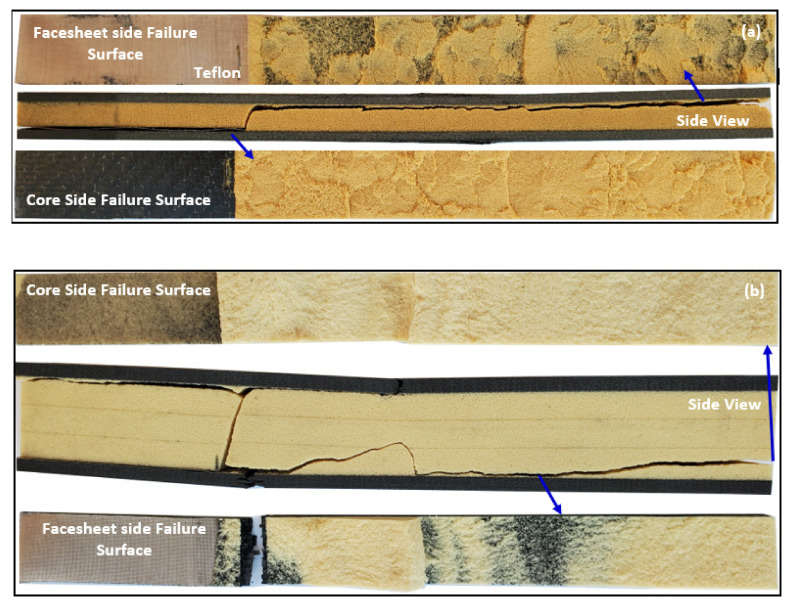
Failure surfaces of posted specimens (**a**): thin cored specimens; (**b**) thick cored specimens.

The surface morphologies of both facesheet and core sided surfaces along the debonded crack from both thin and thick-cored specimens are illustrated in Figure 11. A small visible layer of the polyurethane foam was found to remain attached to the facesheet in both categories of specimens, which was indicative of core failure adjacent to the CFRP facesheet and PU foam rather the complete interfacial failure.

## 5. Conclusions

An experimental investigation was conducted to investigate the flexural response and damage mechanisms of sandwich beams that had a core-facesheet interfacial debond located at one end of the beam. The major contribution of this research article lies in the fact that it sheds light on the failure mechanisms of degraded sandwich beams that have a face-core debond, as such sandwich constructions have not been studied extensively subjected to flexural loadings. The effect of various parameters—such as the foam core thickness, loading rates, and placing the initial interfacial crack under a compressive and tensile stress region—on the flexural failure mechanism of such sandwich beams made from carbon/epoxy facesheets over PU foam was investigated. Under flexural loadings, it was found that the crack tip of the initial debonds between the facesheet and the core in such sandwich beams served as a damage initiation trigger followed by the fracture failure of the core due to the growth of the crack along the height of the sandwich beam and finally the rupture of the facesheet.

Increasing the thickness of the core by 3.2 times resulted in a 220% higher peak load but the first failure of the sandwich beam was observed to occur at 25% lower displacement values. The location of implanted debonds (i.e., placed either in the tension side or compression side) had a negligible effect on the loading capacity of the thin core beams tested under three- or four-point tests, while those had remarkable effects on the load-carrying capacity of thick core beams. In the thick cored beams tested under four-point loading, core shear failure was not observed for sandwich beams that had embedded debonds placed in the compression side, whereas it was the primary failure mode along with upper CFRP facesheet failure underneath loading points for sandwich beams that had an initial pre-crack placed in the tensile side. The peak load value increases by 1200N for thick cored sandwich beams tested under four-point loading that had initial debonding placed in the tension side as compared to being placed on the compressive side. A sandwich beam that had a thick core showed a loading rate-dependent behavior, whereas a negligible effect of loading rate was observed on the thin core beams. An increase of 120% was observed in the peak load value for sandwich beams that had a thick core as the loading rate is increased from 0.05 to 1.5 mm*s^-1^.

## Figures and Tables

**Table 1:** Properties of carbon fiber/epoxy facesheets.

Material	Material Parameter	Symbol	Value	Units
CFRP	Density	ρ	1600 ± 5	(kg/m^3^)
	Longitudinal Modulus	E_11_	81.2 ± 0.5	(GPa)
	Transverse Modulus	E_22_	82.0 ± 0.7	
	In-Plane Poisson’s Ratio	ν_21_	0.056 ± 0.002	
	Shear Modulus in 1–2 Plane	G_12_	5.30 ± 0.06	(GPa)
	Shear Modulus in 2–3 Plane	G_23_	3.30 ± 0.4	
	Longitudinal Compressive Strength	X_c_	715 ± 20	(MPa)
	Transverse Compressive Strength	Y_c_	715 ± 15	
	Longitudinal Tensile Strength	X_t_	828 ± 10	
	Transverse Tensile Strength	Y_t_	828 ± 10	
	In-Plane Shear Strength	S_c_	104 ± 5	

**Table 2:** Mechanical properties of polyurethane foam.

Property Nomenclature /Units	Symbolic Representation and Values	
Density (kg/m^3^)	ρ 248 ± 5	
Tensile modulus (MPa)	Parallel to Rise E_xt_ 171.43 ± 3	Perpendicular to Rise E_yt_ 171.43 ± 5
Compressive modulus (MPa)	E_xc_ 118 ± 2	E_yc_ 118.69 ± 5
Shear modulus (MPa)	G_xz_ 57.81 ± 1	G_xy_ 47.98 ± 1.7
Poisson’s ratio	ν_xy_ 0.30 ± 0.02	ν_xz_ 0.31 ± 0.035
Tensile strength (MPa)	X_t_ 3.82 ± 0.2	Y_t_ 3.82 ± 0.1
Shear strength (MPa)	S_xz_ 2.01 ± 0.5	S_xy_ 1.80 ± 0.2

**Table 3:** Peak force and maximum displacement of both thick and thin cored specimens under four-point flexural loadings.

Serial.No.	Core Thickness 9 mm		Core Thickness 29 mm	
	Peak Force (N)	Maximum. Displacement (mm)	Peak Force (N)	Maximum. Displacement (mm)
1 2 3 4 5	2750 2735 2700 2725 2712	35 38 39 36 38	5750 5740 5738 5745 5747	20 19 19.5 21 18.7
